# Host biomarker-based quantitative rapid tests for detection and treatment monitoring of tuberculosis and COVID-19

**DOI:** 10.1016/j.isci.2022.105873

**Published:** 2022-12-26

**Authors:** Louise Pierneef, Anouk van Hooij, Danielle de Jong, Elisa M. Tjon Kon Fat, Krista E. van Meijgaarden, Elisa Petruccioli, Valentina Vanini, Anna H.E. Roukens, Delia Goletti, Paul L.A.M. Corstjens, Simone A. Joosten, Annemieke Geluk, in collaboration with the BEAT-COVID study group, M.S. Arbous, B.M. van den Berg, S. Cannegieter, C.M. Cobbaert, A. van der Does, J.J.M. van Dongen, J. Eikenboom, M.C.M. Feltkamp, A. Geluk, J.J. Goeman, M. Giera, T. Hankemeier, M.H.M. Heemskerk, P.S. Hiemstra, C.H. Hokke, J.J. Janse, S.P. Jochems, S.A. Joosten, M. Kikkert, L. Lamont, J. Manniën, T.H.M. Ottenhoff, M.R. del Prado, N. Queralt Rosinach, M. Roestenberg, M. Roos, A.H.E. Roukens, H.H. Smits, E.J. Snijder, F.J.T. Staal, L.A. Trouw, R. Tsonaka, A. Verhoeven, L.G. Visser, J.J.C. de Vries, D.J. van Westerloo, J. Wigbers, H.J. van der Wijk, R.C. van Wissen, M. Wuhrer, M. Yazdanbakhsh, M. Zlei

**Affiliations:** 1 Department of Infectious Diseases, Leiden University Medical Center, Leiden, the Netherlands; 2 Department of Cell and Chemical Biology, Leiden University Medical Center, Leiden, the Netherlands; 3 National Institute for Infectious Diseases “L. Spallanzani”, IRCCS, Rome, Italy

**Keywords:** Virology, Bacteriology

## Abstract

Diagnostic services for tuberculosis (TB) are not sufficiently accessible in low-resource settings, where most cases occur, which was aggravated by the COVID-19 pandemic. Early diagnosis of pulmonary TB can reduce transmission. Current TB-diagnostics rely on detection of *Mycobacterium tuberculosis (Mtb)* in sputum requiring costly, time-consuming methods, and trained staff. In this study, quantitative lateral flow (LF) assays were used to measure levels of seven host proteins in sera from pre-COVID-19 TB patients diagnosed in Europe and latently *Mtb-*infected individuals (LTBI), and from COVID-19 patients and healthy controls. Analysis of host proteins showed significantly lower levels in LTBI versus TB (AUC:0 · 94) and discriminated healthy individuals from COVID-19 patients (0 · 99) and severe COVID-19 from TB. Importantly, these host proteins allowed treatment monitoring of both respiratory diseases. This study demonstrates the potential of non-sputum LF assays as adjunct diagnostics and treatment monitoring for COVID-19 and TB based on quantitative detection of multiple host biomarkers.

## Highlights

• Quantitative LFAs were used to assess host biomarkers for TB and COVID-19 diagnosis
• Combined biomarker levels discriminated TB from latent TB and COVID-19
• Host biomarker LFAs can be deployed as adjunct diagnostics within clinical context
• Quantitative LFAs enable treatment response monitoring for TB and COVID-19

## Graphical abstract

**Figure:**
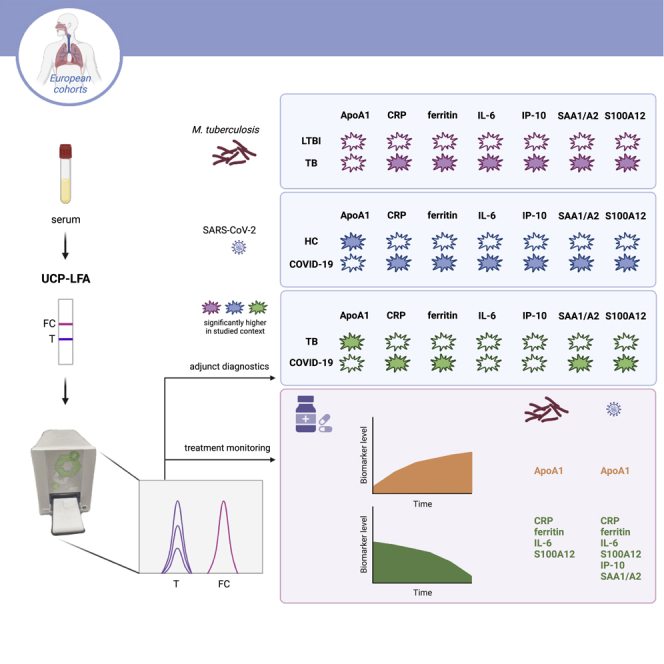

## Introduction

Since the end of 2019, COVID-19, the devastating respiratory disease caused by severe acute respiratory syndrome coronavirus-2 (SARS-CoV-2) has plagued humans, swiftly resulting in a global pandemic. This has led to over 500 million cases and 6 · 3 million deaths (July 2022).^1^ Currently, reverse transcription polymerase chain reaction (RT-PCR) specific for SARS-CoV-2 based on nasopharyngeal swabs are used for diagnosis.^2^ SARS-CoV-2 is transmitted via the respiratory route with an average incubation time of days.^3^ Whereas most individuals infected with this virus are asymptomatic or experience mild to moderate disease,^3^^,^^4^ still a substantial number of patients is hospitalized because of severe respiratory problems.^5^^,^^6^^,^^7^ Moreover, in individuals with severe COVID-19, a proinflammatory cytokine storm can be observed inducing respiratory distress.^4^^,^^8^

Despite the COVID-19 pandemic, tuberculosis (TB) remains one of the most lethal infectious diseases, mainly in low-income countries, but also presenting an unignorable threat in Europe.^1^^,^^9^ In 2020, around 10 million individuals developed TB and 1 · 5 million deaths were attributed to this disease.^9^ It is estimated that one-quarter of the global population is latently infected with *Mycobacterium tuberculosis* (*Mtb*) and approximately 3–10% of those individuals are at risk of developing active TB during their lifetime.^10^ As a poverty-associated disease, TB places a huge burden on health care services of low- and middle-income countries. Of the estimated 10 million patients, 3 · 6 million active TB cases are not diagnosed or reported.^11^ Early diagnosis, followed by prompt and successful treatment will reduce *Mtb* transmission^11^ and prevent disease-associated mortality.^10^ Active TB is diagnosed by detection of the pathogen in sputum using microbiological, microscopic or genetic methods, which are often expensive, time-consuming, resource intense, require specially trained staff, and are less sensitive in HIV co-infected individuals.^12^ Besides, sputum has relatively large sampling error resulting in false negative outcomes as it is difficult to obtain, especially from children.^13^^,^^14^^,^^15^^,^^16^ Also, sputum cultures carry the risk of infection resulting in unusable data. Another hurdle of the use of sputum-based diagnostics is its lack of point-of-care (POC) application, therefore requiring additional clinic visits before treatment initiation. This is associated with a considerable risk of loss to follow-up as this may be a lengthy process, thereby sustaining transmission. TB diagnostic services are not sufficiently accessible, as exemplified by the fact that the WHO endorsed Xpert MTB/RIF (GeneXpert; Cepheid Inc., Sunnyvale, CA, USA) test for TB^17^^,^^18^ is not sufficiently available to people living in remote, TB endemic areas.

Furthermore, it is important to note that COVID-19 contributed to disrupt TB services and therefore local disease control by reducing diagnosis and treatment of active TB and latently *Mtb* infected individuals (LTBI).^19^ Therefore, in the near future, an increase of active TB cases including multidrug-resistant cases, is likely to be observed.^20^^,^^21^^,^^22^ Considering that the risk of death because of TB has been estimated as 1 · 4 times higher in COVID-19 patients,^23^ it is vital to develop new tools to identify patients with TB and/or SARS-CoV-2 infection. As signs and symptoms of COVID-19 might resemble those that are associated with active TB in areas where both are endemic or in “western” settings encountering refugees from TB endemic areas, biomarkers that can rapidly discriminate between these viral and bacterial respiratory diseases can be useful for triage.

Using the luminescent upconverting reporter particle (UCP) technology combined with low-cost, field-friendly immune-chromatography, we have previously developed and field-evaluated quantitative lateral flow assays (LFAs).^24^^,^^25^^,^^26^^,^^27^^,^^28^^,^^29^^,^^30^^,^^31^ These UCP-LFAs are suitable for accurate quantification of cytokines, acute phase proteins and antibodies in serum, stimulated whole blood, pleural fluid, saliva, and fingerstick blood.^29^^,^^32^^,^^33^^,^^34^^,^^35^ The user-friendly, low complexity UCP-LFAs do not require sophisticated analytical laboratory equipment. Portable battery-operated readers provide full instrument-assisted analysis which also avoid potential operator bias. The UCP-LFAs represent POC alternatives for the more elaborate and time consuming, laboratory-based enzyme linked immunosorbent assay (ELISA). Another advantage of the UCP-LFA is that it permits multiplexing to measure several markers simultaneously allowing a biomarker signature to be assessed in field settings.^27^^,^^31^

Exploratory proteomics previously identified a promising host protein signature that distinguished active TB patients from other respiratory diseases (ORD) with signs and symptoms suggestive of TB in an African setting.^36^ Based on this signature, we have applied C-reactive protein (CRP), apolipoprotein-A1 (ApoA1), inducible protein (IP)-10/C-X-C motif chemokine 10 (CXCL-10), and serum amyloid A (SAA) to the UCP-LFA format.^29^ In addition, we have developed UCP-LFAs for interleukin-6 (IL-6), S100 calcium-binding protein A12 (S100A12), and ferritin in view of their role in tuberculous meningitis,^37^^,^^38^ inflammatory disorders,^39^ leprosy,^29^^,^^31^ and iron homeostasis in *Mtb*,^36^^,^^40^ respectively. In this study, we have used UCP-LFAs to rapidly assess serum levels of these host proteins in European TB and COVID-19 patients to investigate to what extent these can identify and discriminate between theold and new respiratory disease.

## Results

### Assessment of host biomarkers for active TB in a European cohort

**Figure 1:**
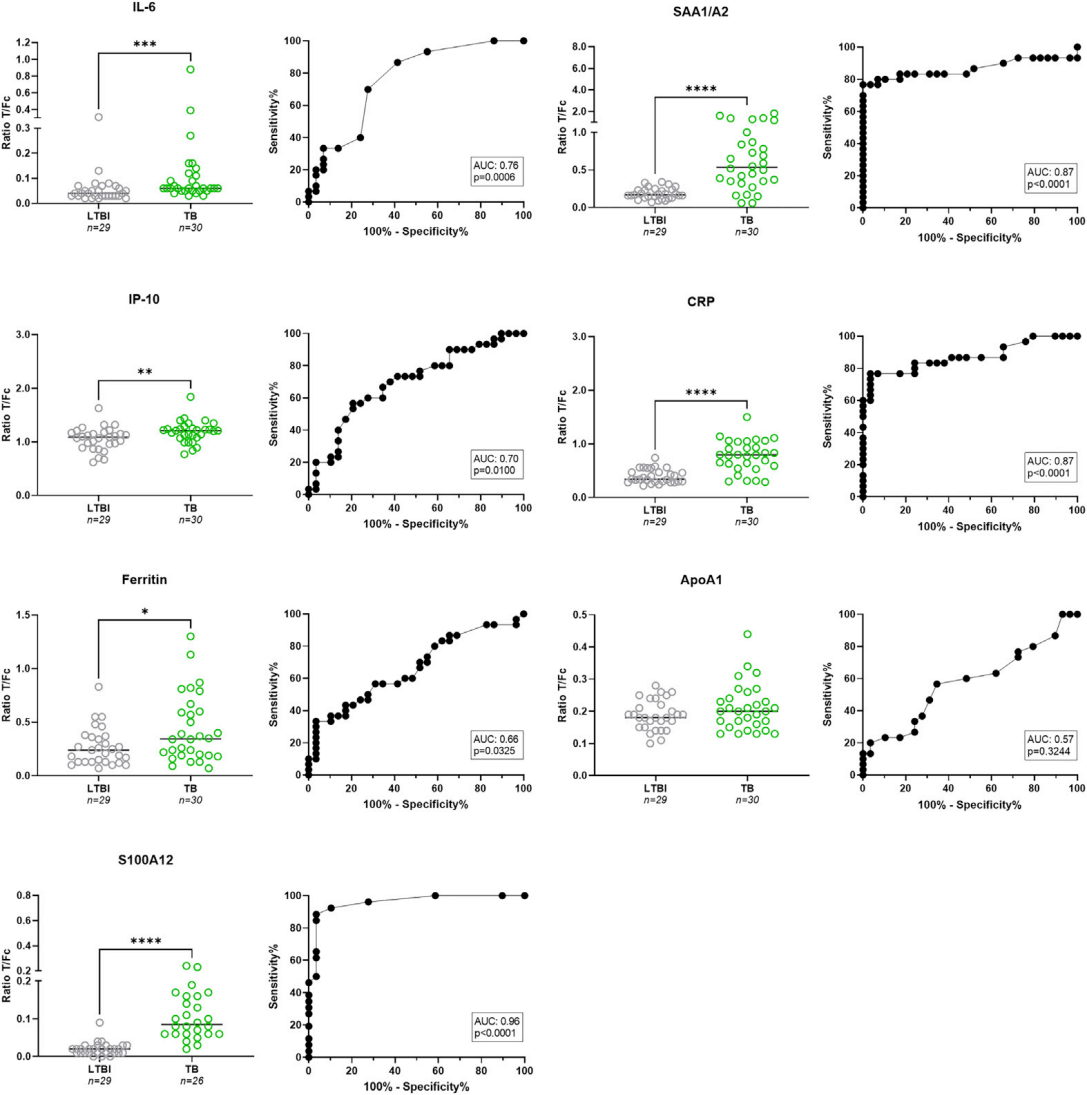
Evaluation of host biomarkers for TB and LTBI in a European cohort Levels of IL-6, IP-10, ferritin, SAA1/A2, CRP, ApoA1, and S100A12 were measured by UCP-LFA in serum samples of TB patients (n = 30; green dots) and LTBI (n = 29; gray dots) from Europe. Median values for each group are indicated by horizontal bars. Mann-Whitney U tests were performed to determine the statistical significance between groups (pvalues: ∗p≤0 · 05, ∗∗p≤0 · 01, ∗∗∗p≤0 · 001, ∗∗∗∗p≤0 · 0001). Green dots: TB cohort 1; gray dots: LTBI cohort 1. AUC: area under the curve; Fc: flow control line; LTBI: latent tuberculosis infection; T: test line; TB: tuberculosis.

Previously, host biomarkers were identified by Luminex as discriminatory between TB and ORD in African settings.^37^ The aim of the present study was to assess whether these host biomarkers could also allow the identification of TB in a European hospital setting, UCP-LF strips were developed for quantitative measurement of seven cytokines^24^^,^^25^^,^^26^^,^^35^^,^^41^^,^^42^ and used for analysis of banked sera from LTBI and pulmonary TB patients collected in Europe (TB cohort 1, Figure 1). Serum levels for CRP, ferritin, IL-6, IP-10, SAA1/A2, and S100A12 were significantly higher in the TB group (p<0 · 0001, p = 0 · 0325, p = 0 · 0006, p = 0 · 01, p<0 · 0001, and p<0 · 0001, respectively), but no significant difference was found for ApoA1 (p = 0 · 3244). CRP, SAA1/A2, and S100A12 were the most discriminatory when comparing TB to LTBI (AUCs: 0 · 87–0 · 96). Chest X-ray severity did not affect the levels for any of the cytokines (Figure S1).

The total number of six biomarkers (ApoA1, CRP, ferritin, IL-6, IP-10, and SAA1/A2) scoring above the cut-off value (NUM score), based on the Youden’s index for each marker, was calculated.^31^ Using a cut-off of ≥3 positive markers, accuracy for active TB (with LTBI as control group) with a sensitivity of 83% (CI: 66 · 4 to 92 · 7) and specificity of 97% (CI: 82 · 8 to 99 · 8) (AUC: 0 · 94; Figure S2) was found. An overview of medians for each marker per test group/comparison and the cut-off values for each biomarker are displayed in Tables 1 and 2. Other NUM score combinations and corresponding AUC and Sn/Sp were evaluated and shown in Table S3. Biomarkers were deleted from the NUM score based on their contribution (AUC); the biomarker with the lowest AUC was removed first and this was repeated until the most discriminatory marker for that comparison remained. Noteworthy is that a 2-marker NUM score of CRP and SAA1/A2 (using a cut-off of ≥1) resulted in an AUC of 0 · 91 and similar Sn/Sp (83%/97%) as the 6-marker NUM score, in the comparison of LTBI versus TB.

**Table 1:** Overview of medians including interquartile range (IQR) for each marker per test group/comparison

**Marker**	**Test group**	**Median**	**IQR**
ApoA1	LTBI cohort 1	0 · 18	0 · 07
	TB cohort 1	0 · 20	0 · 08
	TB cohort 1 + 2	0 · 24	0 · 13
	Healthy controls	0 · 28	0 · 07
	COVID-19	0 · 19	0 · 08
CRP	LTBI cohort 1	0 · 34	0 · 18
	TB cohort 1	0 · 80	0 · 41
	TB cohort 1 + 2	0 · 80	0 · 32
	Healthy controls	0 · 55	0 · 50
	COVID-19	1 · 43	0 · 56
Ferritin	LTBI cohort 1	0 · 24	0 · 24
	TB cohort 1	0 · 35	0 · 40
	TB cohort 1 + 2	0 · 35	0 · 36
	Healthy controls	0 · 06	0 · 09
	COVID-19	0 · 53	0 · 32
IL-6	LTBI cohort 1	0 · 04	0 · 03
	TB cohort 1	0 · 06	0 · 05
	TB cohort 1 + 2	0 · 07	0 · 10
	Healthy controls	0 · 03	0 · 04
	COVID-19	0 · 07	0 · 14
IP-10	LTBI cohort 1	1 · 09	0 · 21
	TB cohort 1	1 · 21	0 · 15
	TB cohort 1 + 2	1 · 11	0 · 33
	Healthy controls	0 · 67	0 · 38
	COVID-19	1 · 02	0 · 60
SAA1/A2	LTBI cohort 1	0 · 17	0 · 09
	TB cohort 1	0 · 54	0 · 51
	TB cohort 1 + 2	0 · 54	0 · 56
	Healthy controls	0 · 25	0 · 21
	COVID-19	1 · 78	0 · 77
S100A12	LTBI cohort 1 + 2	0 · 02	0 · 02
	TB cohort 1	0 · 09	0 · 10
	TB cohort 1 + 2	0 · 07	0 · 08
	Healthy controls	0 · 03	0 · 06
	COVID-19	0 · 17	0 · 16

Overview of the medians including IQR per test group for each individual marker. COVID-19: coronavirus disease 2019; IQR; interquartile range; LTBI: latent tuberculosis infection; TB: tuberculosis.

**Table 2:** Overview of cut-off ratios used for each marker per cohort comparison with corresponding sensitivity and specificity

**Marker**	**Cohort comparison**	**AUC**	**Cut-off ratio**	**Sn/Sp**
ApoA1	TB versus LTBI	*0*·*57*	>0 · 195	57%/66%
	COVID-19 versus healthy controls	0 · 85	<0 · 215	66%/90%
	COVID-19 versus TB	0 · 71	<0 · 285	96%/36%
CRP	TB versus LTBI	0 · 87	>0 · 585	77%/97%
	COVID-19 versus healthy controls	0 · 94	>0 · 990	93%/87%
	COVID-19 versus TB	**0** · **93**	>1 · 175	78%/96%
Ferritin	TB versus LTBI	0 · 66	>0 · 560	33%/97%
	COVID-19 versus healthy controls	0 · 96	>0 · 205	89%/92%
	COVID-19 versus TB	0 · 65	>0 · 405	71%/62%
IL-6	TB versus LTBI	0 · 76	>0 · 045	87%/59%
	COVID-19 versus healthy controls	0 · 72	>0 · 035	75%/67%
	COVID-19 versus TB	*0*·*58*	<0 · 045	34%/92%
IP-10	TB versus LTBI	0 · 70	>1 · 190	57%/79%
	COVID-19 versus healthy controls	0 · 82	>0 · 855	75%/74%
	COVID-19 versus TB	*0*·*55*	>1 · 260	41%/84%
SAA1/A2	TB versus LTBI	0 · 87	>0 · 345	100%/77%
	COVID-19 versus healthy controls	**0** · **98**	>0 · 900	92%/97%
	COVID-19 versus TB	0 · 92	>1 · 005	89%/82%
S100A12	TB versus LTBI	**0 · 96**	>0 · 045	88%/97%
	COVID-19 versus healthy controls	0 · 89	>0 · 125	70%/95%
	COVID-19 versus TB	0 · 79	>0 · 105	76%/70%

Overview of the cut-off ratios used per each individual marker in three different comparisons: TB vs. LTBI, COVID-19 versus healthy controls, and COVID-19 versus TB. The corresponding AUC, sensitivity and specificity are shown for each biomarker. A cut-off ratio for positivity for each biomarker was determined by calculating the maximal Youden’s index. Using the cut-offs for positivity, a NUM score was calculated; the number of biomarkers that scored above the threshold of positivity. AUCs in bold indicate the marker with the most discriminatory potential for a specific comparison; AUCs in italic font indicate a non-significant result. AUC: area under the curve; COVID-19: coronavirus disease 2019; LTBI: latent tuberculosis infection; Sn/Sp: sensitivity/specificity; TB: tuberculosis.

Moreover, comparison of available QuantiFERON data and NUM score results in TB cohort 1 showed that a NUM score based on the levels of six (ApoA1, CRP, ferritin, IL-6, IP-10, and SAA1/A2) as well as two (CRP and SAA1/A2) host proteins, allowed identification of individuals with active TB whose QuantiFERON test was negative (Figure S3). In addition, whereas QuantiFERON was not able to accurately distinguish between active TB and LTBI, both 2- and 6-marker NUM scores successfully discriminated active from latent TB in QuantiFERON-positive individuals.

### Analysis of host proteins for COVID-19 in a European cohort

**Figure 2:**
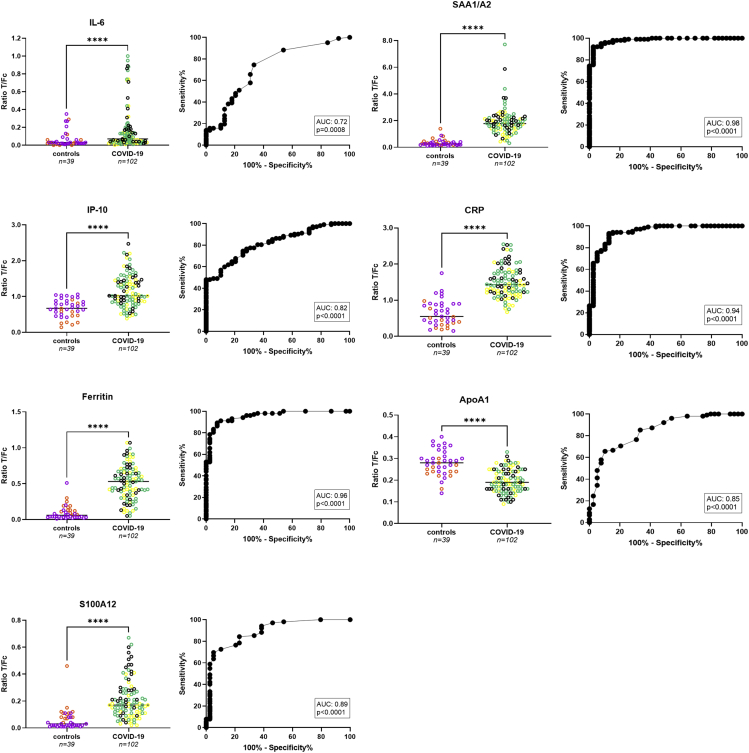
Evaluation of host biomarkers for Dutch COVID-19 patients and healthy controls Levels of IL-6, IP-10, ferritin, SAA1/A2, CRP, ApoA1, and S100A12 were measured by UCP-LFA in serum samples of COVID-19 patients (n = 102) and healthy controls (n = 39; n = 27 from before (May) 2019 (n = 12 from after 2019 (June/July 2020)) from the Netherlands. Median values for each group are indicated by horizontal bars. Mann-Whitney U tests were performed to determine the statistical significance between groups (pvalues: ∗p≤0 · 05, ∗∗p≤0 · 01, ∗∗∗p≤0 · 001, ∗∗∗∗p≤0 · 0001). Orange dots: healthy controls from before 2019; purple dots: healthy controls from after 2019; black dots: COVID-19 patients with a fatal outcome; yellow dots: COVID-19 patients with severe disease; turquoise dots: COVID-19 patients with moderate disease. AUC: area under the curve; COVID-19: coronavirus disease 2019; Fc: flow control line; T: test line.

In 2020, COVID-19 and SARS-CoV-2 infection posed TB diagnostics with an additional respiratory disease in the differential diagnosis. Therefore, UCP-LFA for the same seven biomarkers were also used to analyze hospitalized Dutch COVID-19 patients (n = 102) and healthy controls (n = 39); the latter sampled either in (n = 12) or before 2020 (n = 27; Figure 2). UCP-LFAs showed that for all seven proteins, serum levels were significantly different between the COVID-19 patients and healthy controls, with pvalues ranging from p = 0 · 0008 to p<0 · 0001 (Figure 2). Of note was that for six markers (CRP, ferritin, IL-6, IP-10, SAA1/A2, and S100A12) higher values were detected in COVID-19 sera whereas for ApoA1, significantly lower levels were detected in the COVID-19 group (p<0 · 0001). The markers with the highest discriminatory potential between these two groups included CRP, ferritin, and SAA1/A2 (AUCs ranging from 0 · 94 to 0 · 98). For ApoA1, ferritin, IP-10, and SAA1/A2, no significantly different levels were observed at hospital admission – between COVID-19 patients with moderate disease, severe disease or fatal outcome (Figures 2 and S1). Cytokines CRP, IL-6, and S100A12 were significantly increased in patients with fatal outcome compared to those with moderate disease. In addition, higher CRP levels were observed in severe compared to moderate disease. Moreover, CRP, ferritin, IL-6, IP-10, and SAA1/A2 did not show any significant differences in serum samples at hospital admission of COVID-19 patients who had already received anti-inflammatory treatment before the first sample collection compared to those who had not received any treatment yet (Figure S4). S100A12, on the other hand, significantly decreased on anti-inflammatory treatment, whereas ApoA1 increased.

An in-sample validation of the seven biomarkers (ApoA1, CRP, ferritin, IL-6, IP-10, SAA1/A2, and S100A12) combined through calculation of a NUM score yielded a sensitivity of 93% (CI: 86 · 5 to 96 · 6) with specificity of 100% (CI: 91 · 0 to 100), applying a cut-off of at least four positive markers in the comparison of COVID-19 patients to healthy controls (AUC: 0 · 99; Figure S5; Table 2). Only combining the three most discriminatory markers (CRP, ferritin, and SAA1/A2) resulted in Sn/Sp of 95%/97% (cut-off ≥2) and an AUC of 0 · 99 (Table S3).

### Comparison UCP-LFAs for COVID-19 and TB

**Figure 3:**
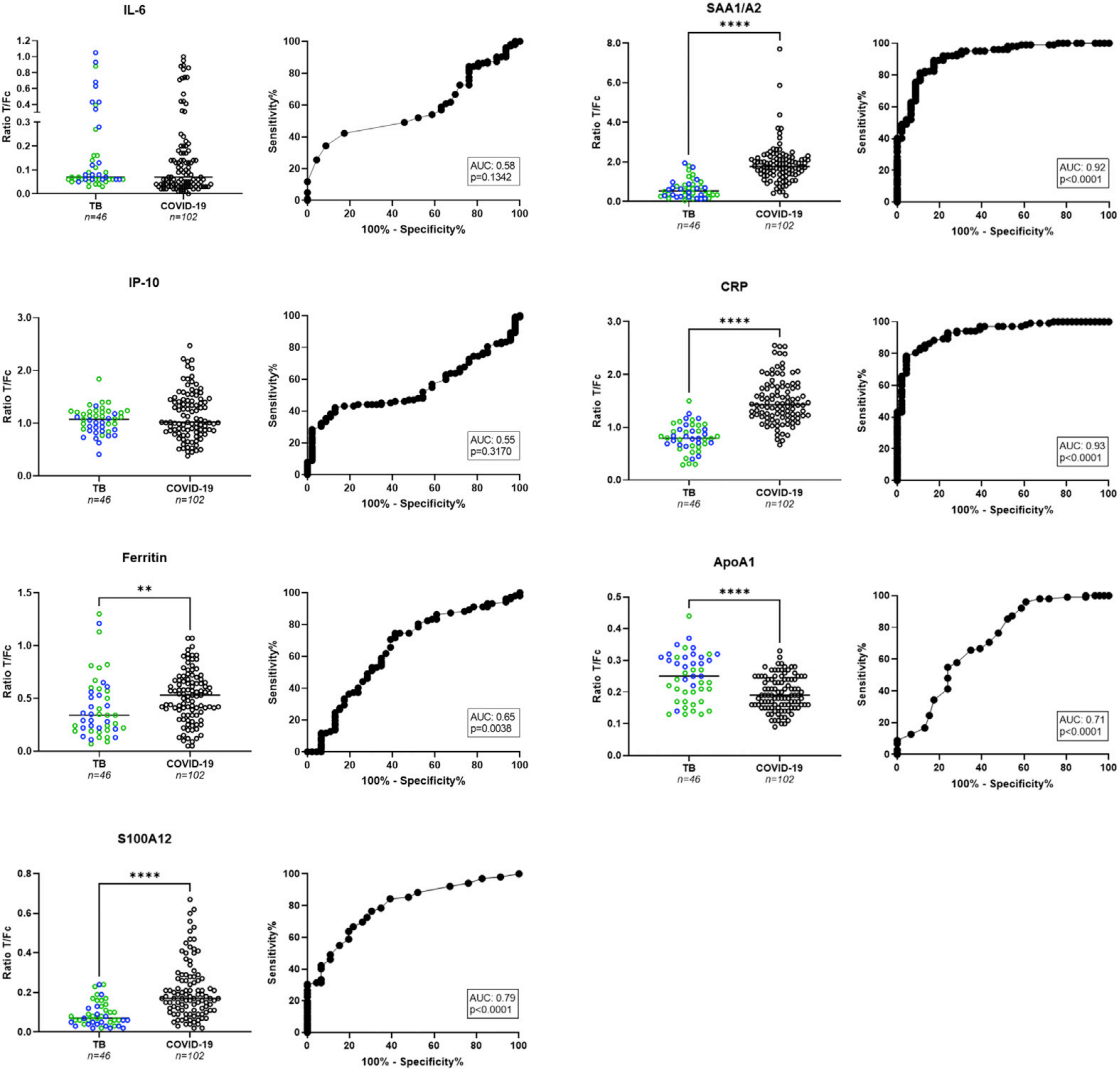
Evaluation of host biomarkers for TB and COVID-19 patients Levels of IL-6, IP-10, ferritin, SAA1/A2, CRP, ApoA1, and S100A12 were measured by UCP-LFA in serum samples of TB patients (n = 46) and COVID-19 patients (n = 102) collected in European hospitals. Median values for each group are indicated by horizontal bars. Mann-Whitney U tests were performed to determine the statistical significance between groups (pvalues: ∗p≤0 · 05, ∗∗p≤0 · 01, ∗∗∗p≤0 · 001, ∗∗∗∗p≤0 · 0001). Green dots: TB cohort 1; blue dots: TB cohort 2; black dots: COVID-19 patients. AUC: area under the curve; COVID-19: coronavirus disease 2019; Fc: flow control line; T: test line; TB: tuberculosis.

To assess whether the seven markers could also distinguish TB from COVID-19, UCP-LFAdata of sera from 26 untreated, pulmonary TB patients collected in Italy (TB cohort 1, Figure 3) were combined with 20 additional sera from former TB patients collected in the Netherlands (TB cohort 2, Figure 3) to increase group size, and compared to 102 serum samples from COVID-19 patients at hospitalization. For CRP, ferritin, SAA1/A2, and S100A12, serum cytokine levels were significantly higher in the COVID-19 group (p<0 · 0001, p = 0 · 0038, p<0 · 0001 and p<0 · 0001, respectively). In contrast, ApoA1 levels were significantly lower in COVID-19 patients (p<0 · 0001). However, no significant differences were found for IL-6 and IP-10 between the two groups. SAA1/A2 and CRP showed the highest discriminatory potential (AUCs of 0 · 92 and 0 · 93, respectively).

When comparing COVID-19 to TB patients, the calculation of a NUM score based on seven biomarkers (ApoA1, CRP, ferritin, IL-6, IP-10, SAA1/A2, and S100A12), resulted in a sensitivity of 91% (CI: 84 · 1 to 95 · 3) with specificity of 87% (CI: 74 · 3 to 93 · 9) applying a cut-off of at least four positive markers (AUC: 0 · 95; Figure S6). A NUM score combining CRP, SAA1/A2, and S100A12 yielded Sn/Sp of 90%/87% applying a cut-off of at least 2 positive markers (AUC: 0 · 94).

### Treatment monitoring

**Figure 4:**
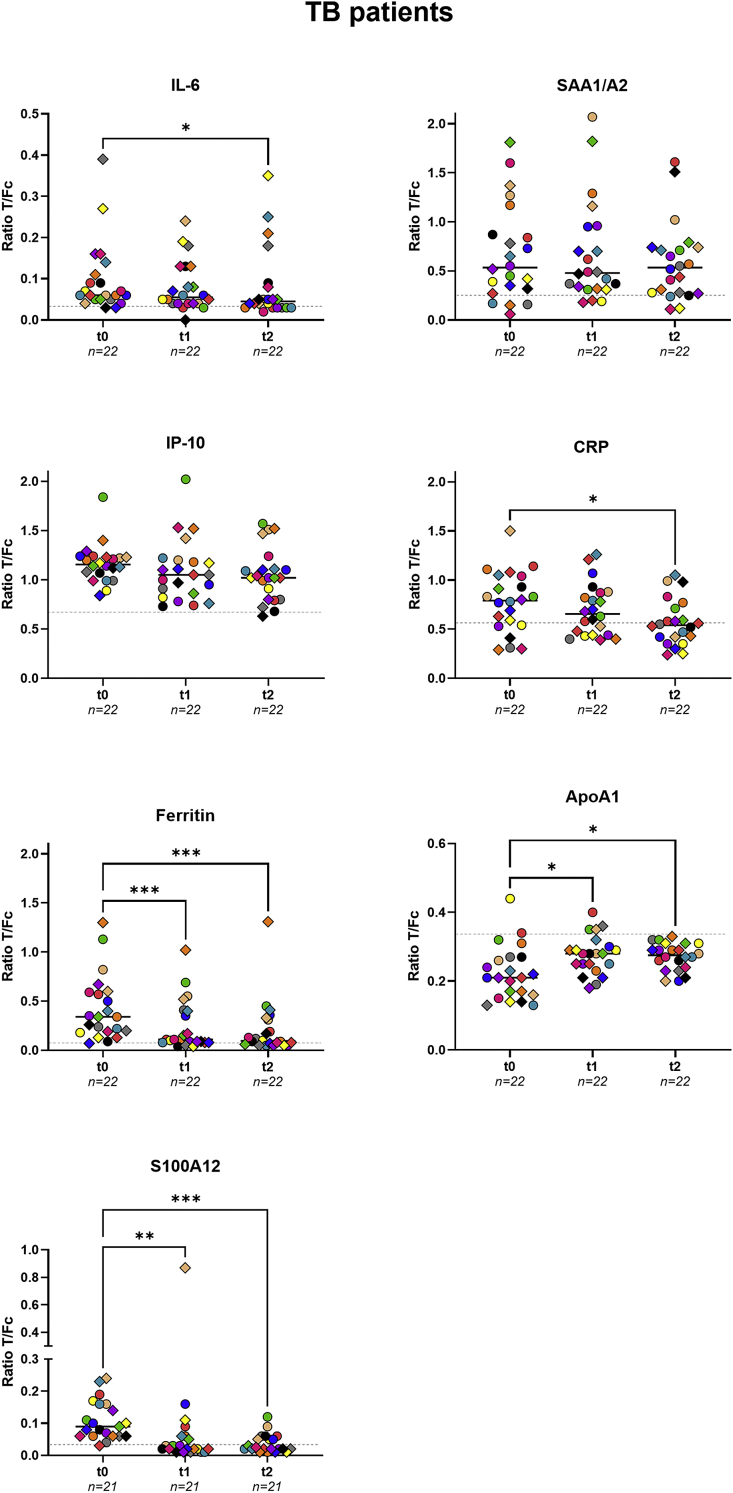
Treatment monitoring for TB Levels of IL-6, IP-10, ferritin, SAA1/A2, CRP, ApoA1, and S100A12 were measured by UCP-LFA in serum samples of pulmonary TB patients (n = 22) before treatment (t_0_), at months 2–4 (t_1_), and months 5–9 (t_2_) of treatment. Median values for each group are indicated by horizontal bars. The gray dotted lines represent the median value of the corresponding marker measured for 39 healthy controls. S100A12 data were missing for one patient. Friedman test with Dunn’s correction for multiple testing was performed to determine the statistical significance between timepoints (pvalues: ∗p≤0 · 05, ∗∗p≤0 · 01, ∗∗∗p≤0 · 001, ∗∗∗∗p≤0 · 0001). Fc: flow control line; T: test line; TB: tuberculosis; t_0_: first timepoints; t_1_: 2–4 months after the beginning of treatment; t_2_: 5–9 months after the beginning of treatment.

To assess the applicability of UCP-LFAs for these markers to monitor TB treatment efficacy, serum samples from 22 confirmed TB patients (from TB cohort 1) taken at month 2–4 (t_1_), and month 5–9 (t_2_) after onset of treatment were analyzed and compared to baseline data before initiation of treatment (t_0_) (Figures 4 and S7). At t_2_, serum levels of IL-6, ferritin, CRP, and S100A12 were significantly reduced compared to participants’ serum levels at t_0_ (p = 0 · 0208, p = 0 · 0002, p = 0 · 0133 and p = 0 · 0004, respectively). In contrast, ApoA1 levels significantly increased at t_2_ compared to t_0_ (p = 0 · 0133), IP-10 and SAA1/A2 showed no significant differences between t_0_ and t_2_ (p = 0 · 1409 and p>0 · 9999, respectively).

**Figure 5:**
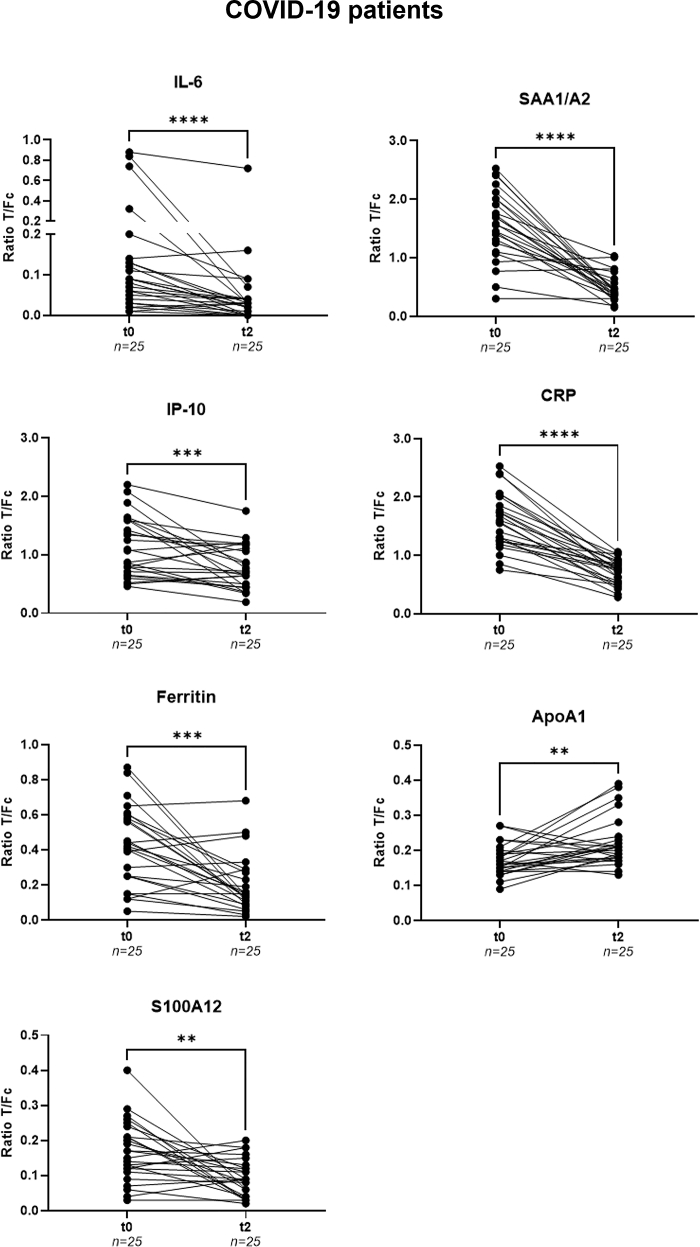
Treatment monitoring for COVID-19 Levels of IL-6, IP-10, ferritin, SAA1/A2, CRP, ApoA1, and S100A12 were measured by UCP-LFA in serum samples from COVID-19 patients (n = 25) at hospital admission (t_0_) and follow-up (t_2_). Median values for each group are indicated by horizontal bars. The gray dotted lines represent the median value of the corresponding marker measured for 39 healthy controls. Wilcoxon matched pairs signed rank tests were performed to determine the statistical significances between timepoints (pvalues: ∗p≤0 · 05, ∗∗p≤0 · 01, ∗∗∗p≤0 · 001, ∗∗∗∗p≤0 · 0001). COVID-19: coronavirus disease 2019; Fc: flow control line; T: test line; t_0_: timepoint of hospital admission; t_2_: follow-up around 6 weeks after hospital discharge.

A similar analysis was performed for 25 successfully treated COVID-19 patients with samples taken at hospital admission (t_0_) and at follow up around 6 weeks after hospital discharge (t_2_ – Figure 5). At t_2_, serum levels of CRP, ferritin, IL-6, IP-10, SAA1/A2, and S100A12 were significantly lower (p<0 · 0001, p = 0 · 0001, p<0 · 0001, p = 0 · 0005, p<0 · 0001 and p = 0 · 0022, respectively). However, ApoA1 levels were significantly higher in comparison to t_0_ (p = 0 · 0063) in line with the finding of reduced ApoA1 serum levels for COVID-19 patients versus healthy controls.

## Discussion

Early detection and treatment of communicable diseases, particularly those spread via aerosols to the respiratory tract, is vital to stop transmission. As shown in WHO records, migrants traveling to Europe may also carry a higher risk of *Mtb* infection.^43^^,^^44^^,^^45^^,^^46^ Thus, in view of the COVID-19 pandemic and the continuous migration to (Western) Europe from areas with multi- and even extensively drug-resistant TB,^47^^,^^48^ specific tools for screening and (rapid) diagnosis of TB, become even more crucial.

This study describes the performance of the rapid and quantitative measurement of seven markers in the UCP-LFA format. This POC platform is highly adaptable toward implementation of the number and variety of biomarkers and was developed for (simultaneous) assessment of cytokines, acute phase proteins,^49^^,^^50^ growth factors,^51^ antibodies, and complement markers.^52^ In this study, LF strips for detection of one biomarker were applied for seven host serum proteins, representing a tentative signature relevant for TB triage, comprising ApoA1, CRP, ferritin, IL-6, IP-10, SAA1/A2, and S100A12.

In comparison to sera from individuals with latent TB, we found significantly increased levels of CRP, ferritin, IL-6, IP-10, SAA1/A2, and S100A12 in those from active TB patients. CRP, SAA, and ferritin, all acute phase proteins (APPs) synthesized in the liver,^53^ are associated with TB^54^^,^^55^^,^^56^^,^^57^^,^^58^^,^^59^^,^^60^ and inflammation.^36^^,^^61^^,^^62^ Once secreted by monocytes, endothelial cells or fibroblasts, IP-10 can act as a chemotactic mediator for both innate and adaptive immune cells,^63^ and is recognized as a marker in HIV-positive TB patients.^64^ IL-6 is a proinflammatory cytokine produced by macrophages.^53^^,^^65^ In line with previous literature, these five markers were also elevated in COVID-19 patients compared to healthy controls.^5^^,^^65^^,^^66^^,^^67^^,^^68^^,^^69^^,^^70^ Furthermore, two additional markers ApoA1 and S100A12, also showed diagnostic potential for COVID-19 confirming the earlier reported downregulation of ApoA1 and the upregulation of S100A12 in COVID-19 patients in France and China.^71^^,^^72^^,^^73^ ApoA1 is believed to play an important role in modulating inflammation as it can inhibit monocyte activation by binding to T cells^74^ resulting in decreased ApoA1 serum levels during inflammation.^75^ On the other hand, the phagocytic protein S100A12 can exhibit proinflammatory effects and is found in high concentrations at sites of inflammation.^39^

When levels of host proteins were compared between COVID-19 and TB patients, five (ApoA1, CRP, ferritin, SAA1/A2, and S100A12) showed promising discriminatory potential as all but ApoA1 were significantly higher in COVID-19 patients’ sera. IL-6 and IP-10 seemed promising biomarkers successfully discriminating TB from LTBI and COVID-19 from healthy controls. However, these two markers did not show potential in distinguishing TB from COVID-19 disease. Although CRP is generally described as a biomarker for bacterial infection,^76^^,^^77^ higher levels of this acute phase protein were observed in COVID-19 patients compared to TB. The excessive levels of these biomarkers could possibly be explained by the acute and exorbitant nature of local and systemic inflammation and immune activation observed in COVID-19 patients.^78^^,^^79^^,^^80^

Despite the fact that certain host proteins are detectable in healthy as well as diseased individuals, the quantitative nature of the UCP-LFA allows the discrimination of TB and COVID-19 at POC because levels varied significantly between the test groups. In this respect, it should be noted that for each comparison (TB versus LTBI; COVID-19 versus HC; TB versus COVID-19) a distinct cut-off was required per biomarker. Besides allowing quantification of host biomarkers at POC, the addition of pathogen-specific biomarkers to a signature based on proteins that are not disease-specific (such as the host proteins evaluated here), increases the diagnostic potential. This was recently demonstrated for leprosy diagnostics in which the simultaneous detection of the cytokines described in this study and anti-*Mycobacterium leprae* PGL-I IgM^29^ into a multi-biomarker test (MBT) allowed the discrimination of patients with both paucibacillary and multibacillary leprosy from controls in high- but also in non-endemic areas.^31^ In the case of *Mtb* infection, however, a specific and sensitive antibody has not yet been identified.^81^^,^^82^

This study aimed to evaluate whether serum levels of the selected host proteins can be used to detect TB and COVID-19 using in-sample validation. These markers need to be validated in an independent cohort, in which both patient groups are recruited prospectively at the same site and time. However, in-sample validation using NUM scores was assessed. This approach, based on serum levels of ApoA1, CRP, ferritin, IL-6, IP-10, and SAA1/A2, yielded an 83% sensitivity and 97% specificity for detection of TB versus LTBI. Noteworthy is that not all six markers might be necessary, as a 4-marker NUM score combining CRP, IL-6, IP-10, and SAA1/A2 in this study yields a sensitivity of 87%, which nears the WHO-recommended sensitivity (90%) for triage TB tests.^83^ Similarly, using a NUM score based on 7 host proteins (additionally including S100A12), COVID-19 patients were separated from healthy controls with sensitivity of 93% and specificity of 100%, respectively. A 3-marker NUM score (CRP, ferritin, and SAA1/A2) resembles the above-mentioned test performance with sensitivity of 95% and specificity of 97%. The seven markers could also distinguish TB from COVID-19 with 91% sensitivity and 87% specificity. A combination of CRP and SAA1/A2 might already be sufficiently discriminatory, with Sn/Sp of 94%/80%.

Longitudinal analysis of both TB and COVID-19 cohorts, indicated that biomarker analysis allows immunomonitoring of treatment for both groups. CRP, ferritin, IL-6, and S100A12 all declined during TB treatment, whereas ApoA1 levels increased over time*.* Furthermore, in line with the use of IL-6 inhibitors for treatment of COVID-19 patients attempting to mediate inflammation,^65^^,^^80^ IL-6 levels declined significantly in these patients on treatment. Of interest, baseline IL-6 levels were significantly higher in patients with fatal outcome compared to those with moderate disease. Nevertheless, contradictory effects on mortality in several clinical trials using IL-6-blocking agents were reported.^65^ Markers described to be valuable in predicting clinical outcome for COVID-19 in other studies included CRP, SAA, ferritin, and S100A12.^62^^,^^69^^,^^73^^,^^84^^,^^85^ For two of those markers, CRP and S100A12, increased levels were indeed detected in our study at hospital admission in patients with fatal outcome compared to those with moderate disease.

Although some patients had been treated with anti-inflammatory medication before hospital admission, this did not affect biomarker levels for CRP, ferritin, IL-6, IP-10, and SAA1/A2. However, only S100A12 and to a lesser extent also ApoA1 already showed a significant effect, arguing for the potential of these proteins as biomarkers for monitoring of early treatment effect.

Our study shows that the UCP-LFA format cannot only provide (adjunct) rapid diagnostics for (triage of) chronic diseases such as TB and leprosy,^31^^,^^34^^,^^36^ but also for more acute diseases including COVID-19. Of note, we demonstrated that in contrast to QuantiFERON-TB Gold which detects *Mtb* infection but cannot discriminate between active TB and LTBI,^86^ the host proteins assessed here showed significant differences between active TB and LTBI (AUC: 0 · 88–1 · 00). Application of these biomarkers in UCP-LFA as adjunct diagnostic tools for triage of TB, can be useful to assess whether further diagnostic testing is warranted thereby reducing the costs for referrals for SARS-CoV-2 PCR and/or GeneXpert.

### Limitations of the study

It should be noted that the COVID-19 cohort studied concerned patients hospitalized in 2020 who were all severely ill but admitted at various COVID-19 stages, which was reflected by the detected range in host biomarker levels. Therefore, in areas endemic for both TB and COVID-19, it will be feasible to triage TB (accepting lower sensitivity) but challenging to diagnose TB based on the studied biomarkers, because these reflect an individual’s disease and inflammation state which may be comparable for these diseases. Consequently, the outcome of non-disease specific, host biomarker-based diagnostics should always be considered in the context of the individual’s clinical presentation and the burden of diseases in an area.

Future studies should thus be focused at simultaneous recruitment of TB as well as all ORD, including other, non-European settings. To this end, the UCP-LFA platform can facilitate replacement of biomarkers to generate more optimal signatures for various use-cases including discrimination of TB and COVID-19.

## Consortium

BEAT-COVID study group: M.S. Arbous, B.M. van den Berg, S. Cannegieter, C.M. Cobbaert, A. van der Does, J.J.M. van Dongen, J. Eikenboom, M.C.M. Feltkamp, A. Geluk, J.J. Goeman, M. Giera, T. Hankemeier, M.H.M. Heemskerk, P.S. Hiemstra, C.H. Hokke, J.J. Janse, S.P. Jochems, S.A. Joosten, M. Kikkert, L. Lamont, J. Manniën, T.H.M. Ottenhoff, M.R. del Prado, N. Queralt Rosinach, M. Roestenberg, M. Roos, A.H.E. Roukens, H.H. Smits, E.J. Snijder, F.J.T. Staal, L.A. Trouw, R. Tsonaka, A. Verhoeven, L.G. Visser, J.J.C. de Vries, D.J. van Westerloo, J. Wigbers, H.J. van der Wijk, R.C. van Wissen, M. Wuhrer, M. Yazdanbakhsh, M. Zlei.

## STAR★Methods

### Key resources table

**Table:** 

REAGENT or RESOURCE	SOURCE	IDENTIFIER
**Antibodies**		

Goat-anti-human ApoA1	R&D systems	AF3664; RRID: AB_2242717
Mouse-anti-human CRP	Labned.com	C5
Mouse-anti-human ferritin	Novus Biologicals	F31
Rat-anti-human IL-6	Biolegend	MQ2-39C3
Mouse-anti-human IP-10	Diaclone Research	B-C55
Mouse-anti-human SAA1/A2	R&D systems	865504
Goat-anti-human S100A12	R&D systems	AF1052; RRID: AB_2183610
Goat-anti-mouse	Sigma-Aldrich	M8642; RRID: AB_260698
Goat-anti-rabbit	Sigma-Aldrich	R4880; RRID: AB_261349
Goat-anti-rat	Sigma-Aldrich	R5130; RRID: AB_261356
Rabbit-anti-goat	Sigma-Aldrich	G4018; RRID: AB_259895
Rabbit-anti-ApoA1	R&D systems	2083A
Mouse-anti-CRP	Labned.com	CRP135
Mouse-anti-ferritin	Novus Biologicals	F23
Mouse-anti-IP-10	Diaclone Research	B-C50
Mouse-anti-SAA1/A2	R&D systems	924903
Goat-anti-S100A12	R&D systems	AF1052; RRID: AB_2183610
Rat-anti-IL-6	Biolegend	MQ2-13A5

**Biological samples**		

Serum samples from individuals with TB and LTBI	the National Institute for Infectious Diseases “L. Spallanzani”, IRCCS, Rome, Italy	
Serum samples from individuals with TB and LTBI	Leiden University Medical Center (LUMC), the Netherlands	
Serum samples from healthy individuals and COVID-19 patients	Leiden University Medical Center (LUMC), the Netherlands	

**Software andalgorithms**		

UCP dedicated benchtop reader	Labrox Oy	Upcon
GraphPad Prism version 9.0.1	GraphPad Software	

**Other**		

Polyacrylic acid functionalized UCPs (200 nm, NaYF_4_:Yb^3+^,Er^3+^)	Intelligent Material Solutions Inc.	

### Resource availability

#### Lead contact

Further information and requests for resources and reagents should be directed to and will be fulfilled by the lead contact, Annemieke Geluk (A.Geluk@lumc.nl).

#### Materials availability

This study did not generate new unique reagents.

### Experimental model and subject details

#### Study participants

Study participants were consenting adults from three different cohorts (Tables S1 and S2):

#### TB cohort 1

Pre-COVID-19 biobanked serum samples from 30 pulmonary TB patients and 29 LTBI patients were recruited at the National Institute for Infectious Diseases “L. Spallanzani”, IRCCS, Rome, Italy, as described earlier.^52^^,^^87^^,^^88^ Pulmonary TB patients were diagnosed based on sputum-culture (BACTEC™, MGIT™, Becton, Dickinson and Company (BD), Franklin Lakes, NJ, USA) or positive Xpert Mtb/RIF assay (Cepheid Inc., Sunnyvale, CA, USA) and included within 7 days after treatment was initiated. LTBI was identified by QuantiFERON-TB Gold-in tube positivity (Qiagen, The Netherlands) in healthysubjects without radiological signs of active disease. All individuals were HIV-negative.

#### TB cohort 2

20 pre-COVID-19 biobanked serum samples were obtained from 20 TB patients during or after treatment recruited at the Leiden University Medical Center (LUMC) in the Netherlands, as described earlier.^89^^,^^90^ TB patients were either HIV-1 seronegative or had no risk factors for exposure to HIV. Ten TB patients were of European origin, three of Asian, and seven of African origin. Since the UCP-LFA for S100A12 was developed at a later point in time than the other six UCP-LFAs, it was evaluated with available LTBI samples from TB cohort 1 (n=11) combined with LTBI samples (n=18) from another European cohort (from TB cohort 2) and compared to 26 TB samples (TB cohort 1).

#### COVID-19 patients from the LUMC BEAT-COVID cohort study

From April 2020 until January 2021, 102 adult participants were admitted to the LUMC with PCR-confirmed SARS-CoV-2 infection and recruited before or during treatment into the LUMC BEAT-COVID cohort study.^91^ Of those 102 individuals, 30 were non-intensive care unit (ICU) patients (moderate disease), 44 were ICU patients (severe disease), and 28 had a fatal outcome due to COVID-19 during hospital stay, as described earlier.^92^ 50 COVID-19 patients had already received anti-inflammatory treatment (i.e. betamethasone, dexamethasone, hydrocortisone, methylprednisolone, and prednisolone) before the first timepoint of blood sample collection, whereas 52 had not. Informed consent was obtained and longitudinal serum sampling was performed for the duration of hospital admission. When possible, convalescent samples were obtained at outpatient follow-up appointments (n=25), around 6 weeks after hospital discharge. All COVID-19 patients were Dutch citizens.

#### Healthy controls

Sera from 39 healthy controls were sampled before (n=27) and during (n=12) the COVID-19 pandemic (June/July 2020) at the LUMC, the Netherlands. The latter were sex (male:female ratio of 2:1) and age-matched to the COVID-19 patients, had no recent history of symptoms of airway infection (fever, cough, hypoxia, rhinorrhea, myalgia, anosmia and/or ageusia or fatigue) and were included in the BEAT-COVID study after confirmed negative SARS-CoV-2-PCR and IgG testing. All healthy controls were Dutch citizens.

#### Ethics

This study was performed according to the Helsinki Declaration (7^th^ revision, 64^th^ Meeting, 2013, Fortaleza). Ethical approval of the study protocol was obtained through the Medical Ethical Committee Leiden-Den Haag-Delft (NL73740.058.20), registered in the Dutch Trial Registry as NL8589 (COVID-19 patients/healthy controls); the Ethical Committee of the L. Spallanzani National Institute of Infectious diseases (INMI; 02/2007 and 72/2015; TB cohort 1); the local Medical Ethics Committee of the Leiden University Medical Center (METC project nr: P07.048 & P207/99; TB cohort 2). Participants were informed about the study objectives, sampling protocol and their right to refuse to take part or withdraw from the study without consequences for their treatment at any point in time. Written informed consent was obtained before enrollment.

### Method details

#### Serum collection

Venous blood samples were collected, in 4 ml plain BD vacutainer serum tubes (BD, Franklin Lakes, NJ, USA). Tubes were centrifuged at 2500 rpm for 10 min and sera were subsequently aliquoted and frozen (–80°C) until use.

#### Lateral flow strips

4 mm width UCP-LF strips specific for a single host protein – ApoA1, CRP, ferritin, IL-6, IP-10, SAA1/A2, and S100A12 - were produced as described earlier.^24^^,^^28^^,^^29^ For ApoA1, CRP, ferritin, IL-6, IP-10, SAA1/A2, and S100A12 LF strips, each Test (T) line comprised 200 ng of the following antibodies: goat-anti-human ApoA1 pAb (AF3664; R&D systems, Minneapolis, MN, USA), mouse-anti-human CRP mAb (C5; Labned.com, Amstelveen, the Netherlands), mouse-anti-human ferritin mAb (F31; Novus Biologicals, Littleton, CO, USA), rat-anti-human IL-6 mAb (MQ2-39C3; Biolegend, San Diego, CA, USA), mouse-anti-human IP-10 mAb (B-C55; Diaclone Research, Besancon, France), mouse-anti-human SAA1/A2 mAb (865504; R&D systems, Minneapolis, MN, USA), and goat-anti-human S100A12 pAb (AF1052; R&D systems, Minneapolis, MN, USA), respectively. To detect non-target bound UCP-conjugated antibodies, Flow-Control (FC) lines comprised 100 ng goat-anti-mouse (M8642; Sigma-Aldrich, St. Louis, MO, USA; for CRP, ferritin, IP-10, and SAA1/A2), goat-anti-rabbit (R4880; Sigma-Aldrich, St. Louis, MO, USA; for ApoA1), goat-anti-rat (R5130; Sigma-Aldrich, St. Louis, MO, USA; for IL-6), or rabbit-anti-goat (G4018; Sigma-Aldrich, St. Louis, MO, USA; for S100A12).

#### UCP conjugates

Antibodies were conjugated to luminescent up-converting reporter particles (UCP) allowing quantitative measurements.^93^^,^^94^^,^^95^ Polyacrylic acid functionalized UCPs (200 nm, NaYF_4_:Yb^3+^,Er ^3+^; Intelligent Material Solutions Inc., Princeton, NJ, USA) were conjugated according to previously described protocols.^96^ Rabbit-anti-ApoA1 (2083A; R&D systems, Minneapolis, MN, USA), mouse-anti-CRP (CRP135; Labned.com, Amstelveen, Netherlands), mouse-anti-ferritin (F23; Novus Biologicals, Littleton, CO, USA), mouse-anti-IP-10 (B-C50; Diaclone Research, Besancon, France), mouse-anti-SAA1/A2 (924903; R&D systems, Minneapolis, MN, USA), and goat-anti-S100A12 (AF1052; R&D systems, Minneapolis, MN, USA) were bound at a concentration of 50 μg antibody per mg UCP. Rat-anti-IL-6 (MQ2-13A5; Biolegend, San Diego, CA, USA) was bound at a concentration of 25 μg per mg UCP. Stock solutions were kept at 4°C until use. UCPs were incorporated in the sample/conjugate pad at a density of 200 ng per 4 mm (ApoA1, CRP, ferritin, IL-6, SAA1/A2, and S100A12) or 400 ng per 4 mm (IP-10).

#### UCP-LFA

10-fold (ferritin, IL-6, and IP-10), 100-fold (S100A12), 1,000-fold (CRP and SAA1/A2), and 10,000-fold (ApoA1) serum dilutions were prepared in high salt buffer (100mM Tris pH 8, 270 mM NaCl, 1% (v/v) Triton X-100, 1% (w/v) BSA). 100 μl of diluted serum samples was added to a 96-wells plate and LF was initiated by placing the UCP-LF strip into the well. Immunochromatography was allowed to continue until strips were dry. UCP-LF strips were scanned with a UCP dedicated benchtop reader (UPCON; Labrox Oy Turku, Finland). Results are calculated as the ratio value (R) between Test and Flow Control signal based on relative fluorescence units (RFUs) measured at the respective lines.

### Quantification and statistical analysis

GraphPad Prism version 9.0.1 for Windows (GraphPad Software, San Diego, CA, USA) was used to perform statistical analysis. Mann-Whitney U tests and Kruskal-Wallis tests were performed to determine the statistical significance between two and three independent groups, respectively. Wilcoxon matched pairs signed rank tests and Friedman tests with Dunn’s correction were performed to determine the statistical significance between two and three paired timepoints, respectively. Plot receiver operating characteristic (ROC) curves were created and sensitivity (Sn), specificity (Sp) and the area under the curve (AUC) were calculated to evaluate test performance. A cut-off for positivity for each biomarker was determined by calculating the maximal Youden’s index.^97^

For each of the individuals tested, an extra parameter (NUM score),^31^ was calculated, representing the number of biomarkers that scored above the threshold of positivity based on the Youden’s index. Three comparisons were made: TB vs. LTBI, COVID-19 vs. healthy controls, and COVID-19 vs. TB. For each comparison, a NUM score was calculated with the number of biomarkers used ranging from 1 to 7.

## Data Availability

• All biomarker data reported in this paper will be shared by the lead contact upon request. • This paper does not report original code. • Any additional information required to reanalyze the data reported in this paper is available from the lead contact upon request.
