# Supplementary material for: Host biomarker-based quantitative rapid tests for detection and treatment monitoring of tuberculosis and COVID-19
Source: iScience. 2022 Dec 26;26(1):105873. doi: 10.1016/j.isci.2022.105873 (PMC9791715; doi:10.1016/j.isci.2022.105873)
Supplement: Document S1. Figures S1–S7 and Tables S1–S3 [file mmc1.pdf]

iScience, Volume *26*

Supplemental information

Host biomarker-based quantitative rapid tests for detection and treatment monitoring of tuberculosis and COVID-19

Louise Pierneef, Anouk van Hooij, Danielle de Jong, Elisa M. Tjon Kon Fat, Krista E. van Meijgaarden, Elisa Petruccioli, Valentina Vanini, Anna H.E. Roukens, Delia Goletti, Paul L.A.M. Corstjens, Simone A. Joosten, Annemieke Geluk, and in collaboration with the BEAT-COVID study group

SUPPLEMENTAL INFORMATION

SUPPLEMENTARY TABLE S1 ***Description of the cohorts, Related to Figures 1-4 and Tables 1-2***

**Cohort**	**Country of sampling**	**Group**	***N***	**Age mean (range)**	**Sex (% female)**
TB cohort 1	Italy	TB	30	34 (18 – 52)	43
		LTBI	29	45 (19 – 75)	69
TB cohort 2	The Netherlands	TB	20	44 (15 – 84)	20
		LTBI	18	35 (18 – 60)	61
COVID-19/healthy controls	The Netherlands	COVID-19	102	63 (18 – 91)	23
		healthy controls	39	42 (22 – 72)	59
		pre-COVID-19	*27*	*33 (22 – 60)*	*70*
		during COVID-19	*12*	*64 (60 – 72)*	*33*

Overview of the different cohorts including country of sampling, group, number of samples per group, age, and sex. COVID-19: coronavirus disease 2019; LTBI: latent tuberculosis infection; TB: tuberculosis.

Supplementary Table S2	*Country of origin of the participants, Related to Figures 1-4 and Tables 1-2*		
**Cohort**	**Group**	**Country of origin**	***N***
TB cohort 1	TB	*Albania*	2
		Colombia	1
		Georgia	1
		Italy	10
		Kosovo	1
		Moldova	1
		Romania	14
	LTBI	*Afghanistan*	1
		Colombia	1
		Italy	20
		Lithuania	1
		Peru	1
		Romania	5
TB cohort 2	TB	*Angola*	2
		Italy	1
		Jakarta	2
		Morocco	2
		the Netherlands	9
		Somalia	2
		South Africa	1
		Sri Lanka	1
	LTBI	*France*	1
		the Netherlands	14
		Netherlands Antilles	1
		Sri Lanka	2
COVID-19/healthy controls		*Dutch citizens**	141

Overview of the country of origin for the participants in the different cohorts. *Individuals from the COVID-19/healthy controls cohort were all Dutch citizens. COVID-19: coronavirus disease 2019; LTBI: latent tuberculosis infection; TB: tuberculosis.

**Supplementary Table S3**	***Overview of different NUM scores per cohort comparison with corresponding AUC, sensitivity, and specificity, Related to Figures 1-3 and Table 2***				
**Cohort comparison**	**# of markers used in NUM score**	**Markers used in NUM score**	**AUC**	**Cut-off**	**Sn/Sp**
TB vs. LTBI	6	ApoA1, CRP, ferritin, IL-6, IP-10, SAA1/A2	0·94	≥3	83%/97%
	5	CRP, ferritin, IL-6, IP-10, SAA1/A2	0·93	≥2	87%/86%
	4	CRP, IL-6, IP-10, SAA1/A2	0·93	≥2	87%/86%
	3	CRP, IL-6, SAA1/A2	0·94	≥2	80%/97%
	2	CRP, SAA1/A2	0·91	≥1	83%/97%
	1	CRP*	0·87	≥1	77%/97%
	1	SAA1/A2*	0·87	≥1	100%/77%
COVID-19 vs. healthy controls	7	ApoA1, CRP, ferritin, IL-6, IP-10, SAA1/A2, S100A12	0·99	≥4	93%/100%
	6	ApoA1, CRP, ferritin, IP-10, SAA1/A2, S100A12	1·00	≥3	98%/100%
	5	ApoA1, CRP, ferritin, SAA1/A2, S100A12	1·00	≥3	95%/100%
	4	CRP, ferritin, SAA1/A2, S100A12	0·99	≥2	96%/97%
	3	CRP, ferritin, SAA1/A2	0·99	≥2	95%/97%
	2	ferritin, SAA1/A2	0·98	≥1	99%/92%
	1	SAA1/A2	0·98	≥1	92%/97%
COVID-19 vs. TB	7	ApoA1, CRP, ferritin, IL-6, IP-10, SAA1/A2, S100A12	0·95	≥4	91%/87%
	6	ApoA1, CRP, ferritin, IL-6, SAA1/A2, S100A12	0·95	≥3	98%/83%
	5	ApoA1, CRP, ferritin, SAA1/A2, S100A12	0·94	≥3	93%/83%
	4	ApoA1, CRP, SAA1/A2, S100A12	0·95	≥3	88%/89%
	3	CRP, SAA1/A2, S100A12	0·94	≥2	90%/87%
	2	CRP, SAA1/A2	0·93	≥1	94%/80%
	1	CRP	0·93	≥1	78%/96%

Overview of the different NUM scores per cohort comparison with corresponding AUC, sensitivity, and specificity. A cut-off ratio for positivity for each biomarker was determined by calculating the maximal Youden's index. Using the cut-offs for positivity, a NUM score was calculated; the number of biomarkers that scored above the threshold of positivity. Three comparisons were made: TB vs. LTBI, COVID-19 vs. healthy controls, and COVID-19 vs. TB. Biomarkers were deleted from the NUM score based on their contribution (AUC); the biomarker with the lowest AUC was removed first, ending up with the most discriminatory marker for that comparison. *Since CRP and SAA1/A2 both showed an AUC of 0·87 in the comparison of TB vs. LTBI, both options as a final marker remaining are shown. AUC: area under the curve; COVID-19: coronavirus disease 2019; LTBI: latent tuberculosis infection; Sn/Sp: sensitivity/specificity; TB: tuberculosis.

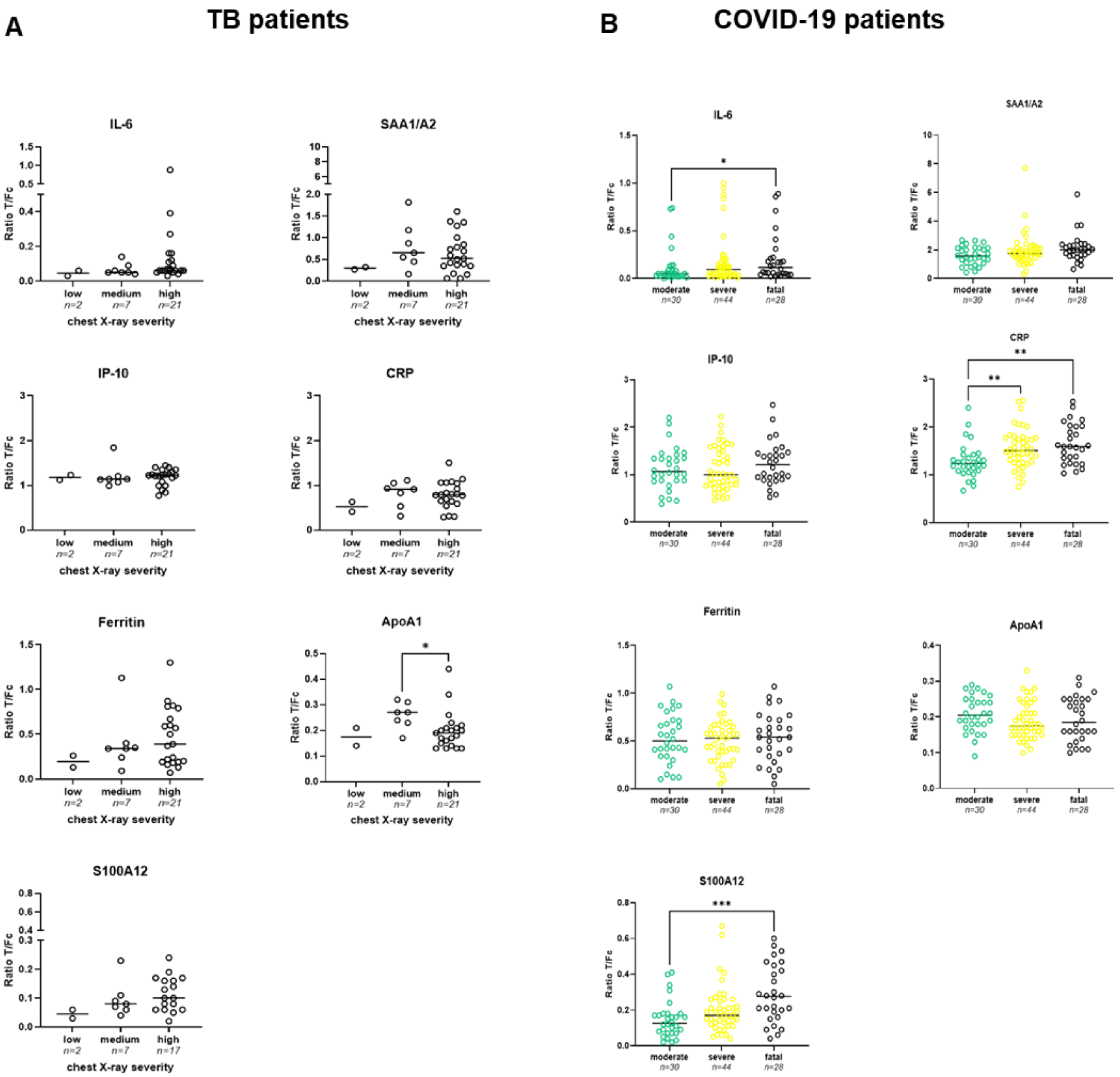
A
TB patients
B
COVID-19 patients
IL-6
SAA1/A2
IP-10
CRP
Ferritin
ApoA1
S100A12
Ratio T/Fc
low
medium
high
n=2
n=7
n=21
n=17
chest X-ray severity
moderate
severe
fatal
n=30
n=44
n=28
*
**
***

SUPPLEMENTARY FIGURE S1: ***Evaluation of seven biomarkers for disease severity of TB and COVID-19, Related to Figures 1-2 and Table 1***

ApoA1, CRP, ferritin, IL-6, IP-10, SAA1/A2, and S100A12 were measured by singleplex strips in serum samples of TB patients with low (n=2), medium (n=7) and high (n=21) chest X-ray severity (A) and serum samples at hospital admission of COVID-19 patients with ultimate moderate disease (n=30), severe disease (n=44), and fatal outcome (n=28) from the Netherlands (B). Median values for each group are indicated by horizontal bars. A Kruskal-Wallis test was performed to determine the statistical significance between groups (p-values: *p≤0·05, **p≤0·01, ***p≤0·001, ****p≤0·0001). Green dots: COVID-19 patients with moderate disease, yellow dots: COVID-19 patients with severe disease; black dots: COVID-19 patients with a fatal outcome. COVID-19: coronavirus disease 2019; Fc: flow control line; T: test line; TB: tuberculosis.

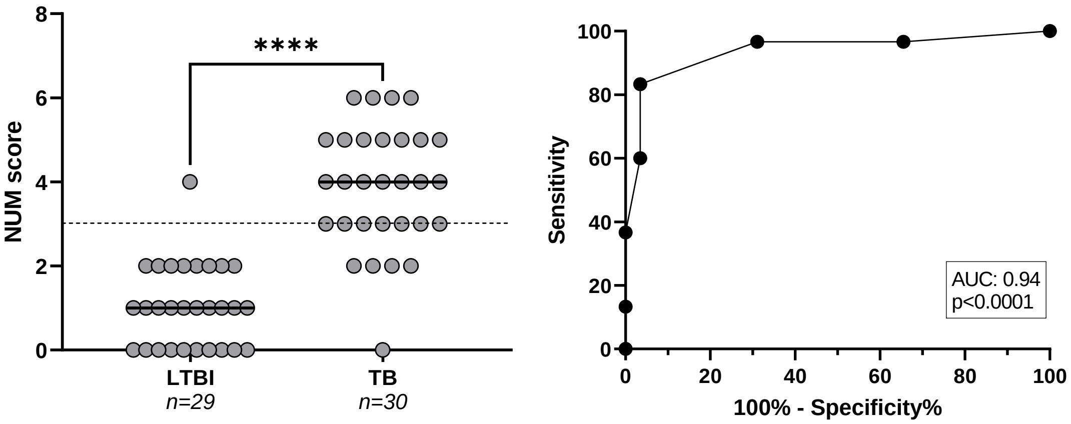

Cut-off	Sensitivity (%)	95% CI	Specificity (%)	95% CI
≥ 1	97	83·3% to 99·8%	34	19·9% to 52·7%
≥ 2	97	83·3% to 99·8%	69	50·8% to 82·7%
**≥ 3**	**83**	**66·4% to 92·7%**	**97**	**82·8% to 99·8%**
≥ 4	60	42·3% to 75·4%	97	82·8% to 99·8%
≥ 5	37	21·9% to 54·5%	100	88·3% to 100%
≥ 6	13	5·3% to 29·7%	100	88·3% to 100%

SUPPLEMENTARY FIGURE S2: ***NUM score evaluation of six biomarkers for TB and LTBI in European cohort, Related to Figure 1***

ApoA1, CRP, ferritin, IL-6, IP-10, and SAA1/A2 were measured by singleplex strips in serum samples of TB patients (n=30) and LTBI (n=29) from Europe. The NUM score (the number of positive biomarkers) is indicated on the *y*-axis. Median values for each group are indicated by horizontal bars. A Mann-Whitney U test was performed to determine the statistical significance between groups (p-values: *p≤0·05, **p≤0·01, ***p≤0·001, ****p≤0·0001). The cut-off with optimal sensitivity and specificity is written in bold and depicted as a dotted horizontal line in the graph. AUC: area under the curve; CI: confidence interval; LTBI: latent tuberculosis infection; TB: tuberculosis.

A 6-marker NUM score

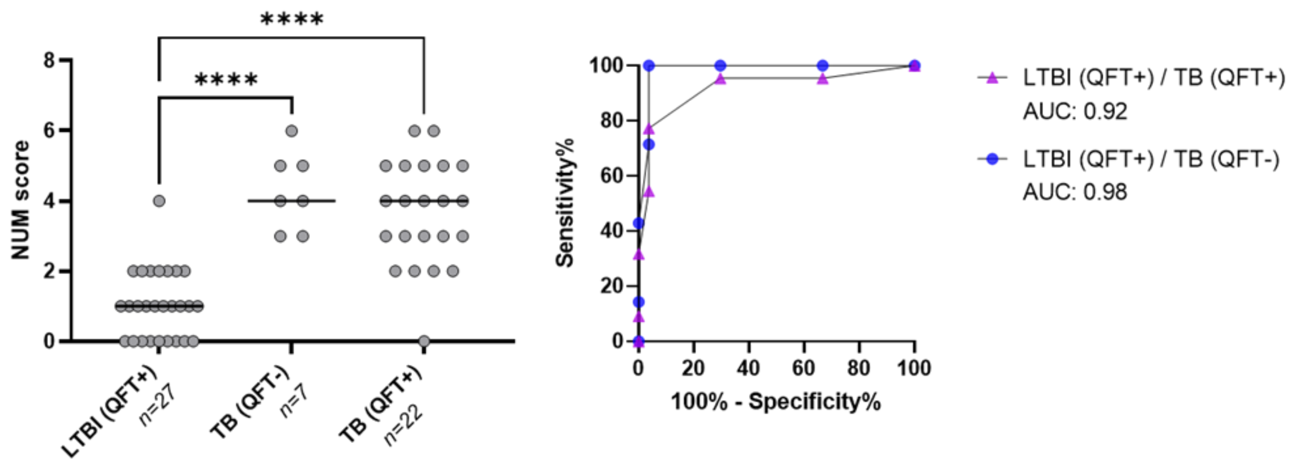

B 2-marker NUM score

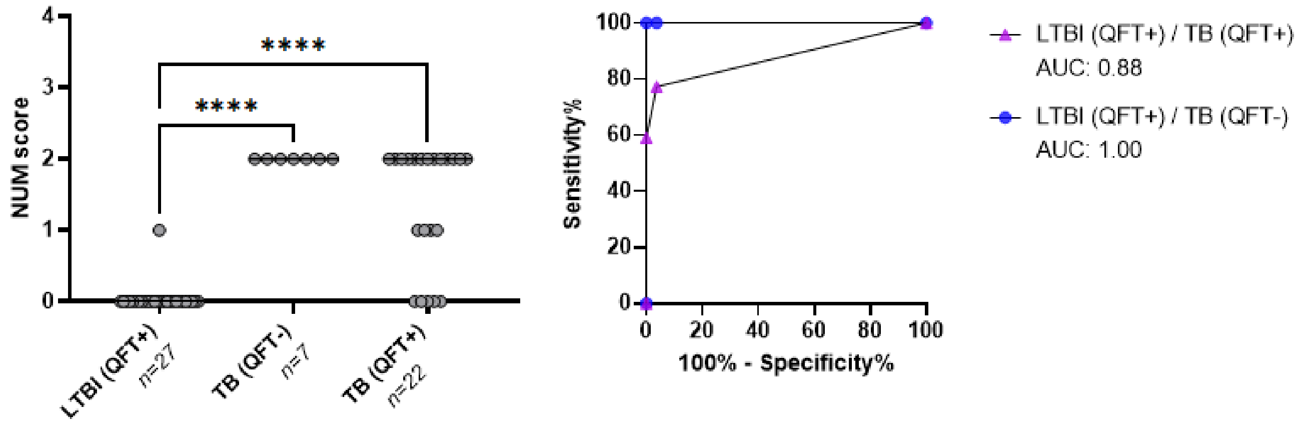

SUPPLEMENTARY FIGURE S3: ***Comparison of QuantiFERON and NUM score results for TB and LTBI patients, Related to Figure 1***

ApoA1, CRP, ferritin, IL-6, IP-10, and SAA1/A2 were measured by singleplex strips in serum samples of: QuantiFERON-negative TB patients (n=7) and QuantiFERON-positive LTBI (n=27) and TB (n=22) patients from Europe. The NUM score (the number of positive biomarkers) is indicated on the *y*-axis; ApoA1, CRP, ferritin, IL-6, IP-10, and SAA1/A2 were combined into a 6-marker NUM score (A) and CRP and SAA1/A2 were combined into a 2-marker NUM score (B). Median values for each group are indicated by horizontal bars. A

Kruskal-Wallis test was performed to determine the statistical significance between groups (p-values: *p≤0·05, **p≤0·01, ***p≤0·001, ****p≤0·0001). AUC: area under the curve; LTBI: latent tuberculosis infection; QFT: QuantiFERON; TB: tuberculosis.

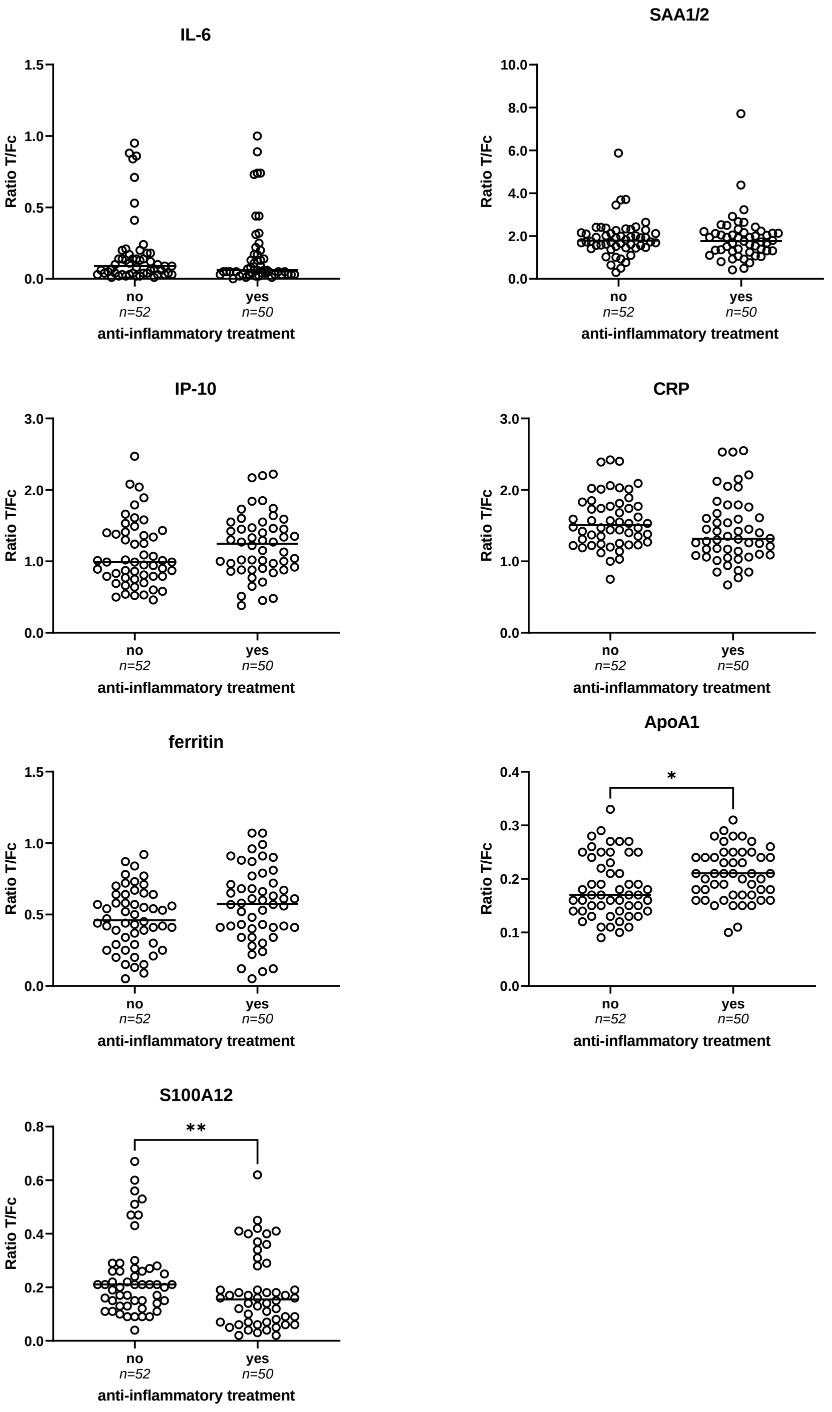

SUPPLEMENTARY FIGURE S4: ***Effect of anti-inflammatory treatment on seven host proteins in COVID-19 patients, Related to Figure 2***

ApoA1, CRP, ferritin, IL-6, IP-10, SAA1/A2, and S100A12 were measured by singleplex strips in serum samples at hospital admission of COVID-19 patients which had already received anti-inflammatory treatment before the first sample collection (n=50) and those who

had not (n=52). Anti-inflammatory treatment included betamethasone, dexamethasone, hydrocortisone, methylprednisolone or prednisolone. Median values for each group are indicated by horizontal bars. A Mann-Whitney U test was performed to determine the statistical significance between groups (p-values: *p≤0·05, **p≤0·01, ***p≤0·001, ****p≤0·0001). Fc; flow control line; T: test line.

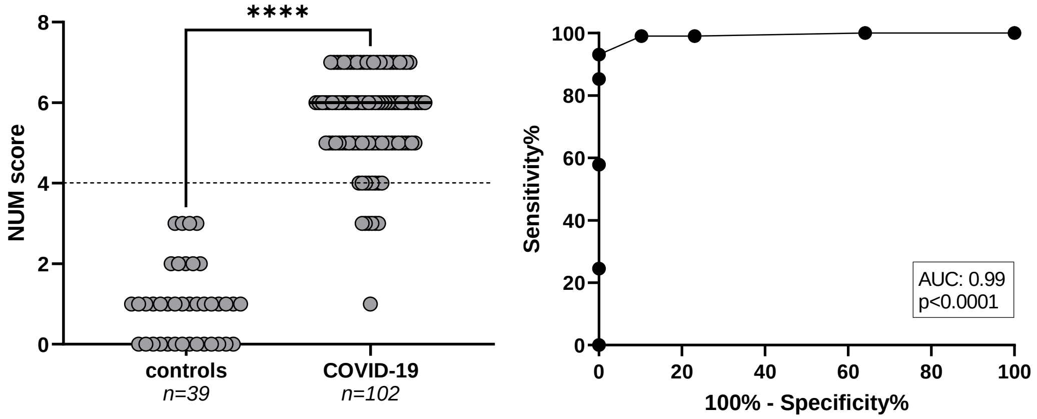

Cut-off	Sensitivity (%)	95% CI	Specificity (%)	95% CI
≥ 1	100	96·4% to 100%	36	22·7% to 51·6%
≥ 2	99	94·7% to 100%	77	61·7% to 87·4%
≥ 3	99	94·7% to 100%	90	76·4% to 95·9%
**≥ 4**	**93**	**86·5% to 96·6%**	**100**	**91·0% to 100%**
≥ 5	85	77·2% to 90·9%	100	91·0% to 100%
≥ 6	58	48·2% to 67·0%	100	91·0% to 100%
≥ 7	25	17·2% to 33·7%	100	91·0% to 100%

SUPPLEMENTARY FIGURE S5: ***NUM score evaluation of seven biomarkers for healthy controls and COVID-19 patients from the Netherlands, Related to Figure 2***

ApoA1, CRP, ferritin, IL-6, IP-10, SAA1/A2, and S100A12 were measured by singleplex strips in serum samples of controls (n=39) and COVID-19 patients (n=102) from the Netherlands. The NUM score (the number of positive biomarkers) is indicated on the *y*-axis. Median values for each group are indicated by horizontal bars. A Mann-Whitney U test was performed to determine the statistical significance between groups (p-values: *p≤0·05, **p≤0·01, ***p≤0·001, ****p≤0·0001). The cut-off with optimal sensitivity and specificity is written in bold and depicted as a dotted horizontal line in the graph. AUC: area under the curve; CI: confidence interval; COVID-19: coronavirus disease 2019.

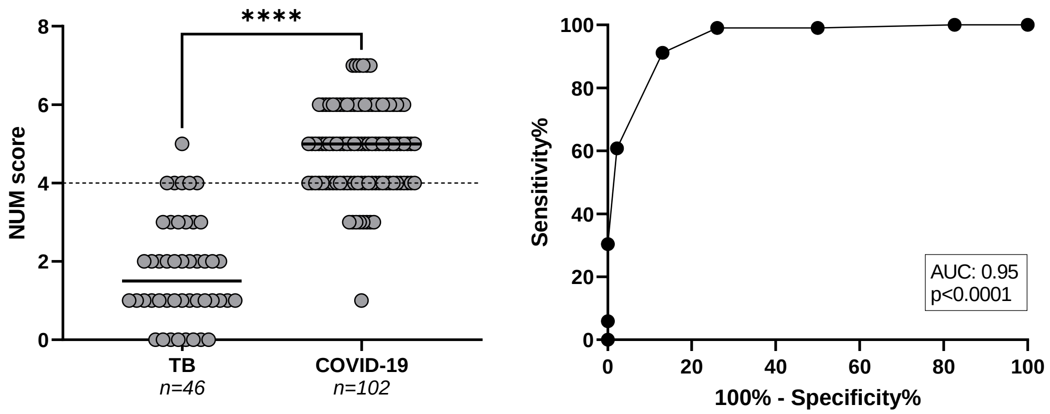

Cut-off	Sensitivity (%)	95% CI	Specificity (%)	95% CI
≥ 1	100	96·4% to 100%	17	9·1% to 30·7%
≥ 2	99	94·7% to 100%	50	36·1% to 63·9%
≥ 3	99	94·7% to 100%	74	59·7% to 84·4%
**≥ 4**	**91**	**84·1% to 95·3%**	**87**	**74·3% to 93·9%**
≥ 5	61	51·1% to 69·7%	98	88·7% to 99·9%
≥ 6	30	22·3% to 39·9%	100	92·3% to 100%
≥ 7	6	2·7% to 12·2%	100	92·3% to 100%

SUPPLEMENTARY FIGURE S6: ***NUM score evaluation of seven biomarkers for TB and COVID-19 patients, Related to Figure 3***

ApoA1, CRP, ferritin, IL-6, IP-10, SAA1/A2, and S100A12 were measured by singleplex strips in serum samples of TB patients (n=46) from Europe and COVID-19 patients (n=102) from the Netherlands. The NUM score (the number of positive biomarkers) is indicated on the *y*-axis. Median values for each group are indicated by horizontal bars. A Mann-Whitney U test was performed to determine the statistical significance between groups (p-values: *p≤0·05, **p≤0·01, ***p≤0·001, ****p≤0·0001). The cut-off with optimal sensitivity and specificity is written in bold and depicted as a dotted horizontal line in the graph. AUC: area under the curve; CI: confidence interval; COVID-19: coronavirus disease 2019; TB: tuberculosis.

TB patients

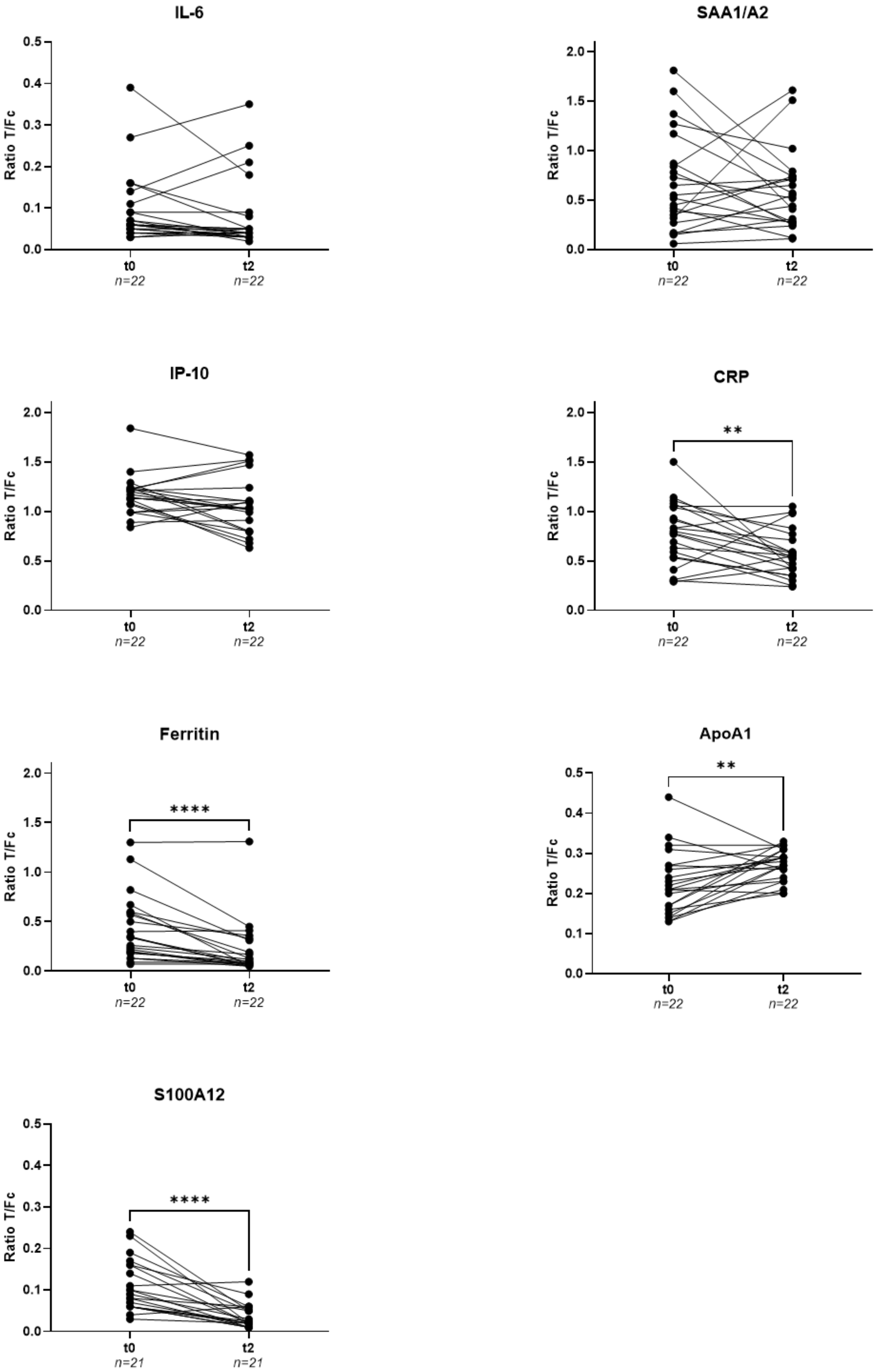
IL-6
Ratio T/Fc
0.0
0.1
0.2
0.3
0.4
0.5
t0
n=22
t2
n=22
SAA1/A2
Ratio T/Fc
0.0
0.5
1.0
1.5
2.0
t0
n=22
t2
n=22
IP-10
Ratio T/Fc
0.0
0.5
1.0
1.5
2.0
t0
n=22
t2
n=22
CRP
**
Ratio T/Fc
0.0
0.5
1.0
1.5
2.0
t0
n=22
t2
n=22
Ferritin
****
Ratio T/Fc
0.0
0.5
1.0
1.5
2.0
t0
n=22
t2
n=22
ApoA1
**
Ratio T/Fc
0.0
0.1
0.2
0.3
0.4
0.5
t0
n=22
t2
n=22
S100A12
****
Ratio T/Fc
0.0
0.1
0.2
0.3
0.4
0.5
t0
n=21
t2
n=21

SUPPLEMENTARY FIGURE S7: ***Paired visualization of treatment monitoring for TB, Related to Figure 4***

Treatment monitoring of TB patients (n=22) over time. S100A12 data were missing for one patient. Levels of IL-6, IP-10, ferritin, SAA1/A2, CRP, ApoA1, and S100A12 were measured by UCP-LFA in serum samples of pulmonary TB patients before treatment (t_0), and months 5 – 9 (t_2). Wilcoxon matched pairs signed rank tests were performed to determine the statistical significance between timepoints (p-values: *p≤0·05, **p≤0·01, ***p≤0·001, ****p≤0·0001). Fc: flow control line; T: test line; TB: tuberculosis.
